# Exploring the inhibition mechanism of adenylyl cyclase type 5 by n-terminal myristoylated Gα_i1_

**DOI:** 10.1371/journal.pcbi.1005673

**Published:** 2017-09-11

**Authors:** Siri Camee van Keulen, Ursula Rothlisberger

**Affiliations:** Institut des Sciences et Ingénierie Chimiques, École Polytechnicque Fédérale de Lausanne (EPFL), CH-1015 Lausanne, Switzerland; University of Houston, UNITED STATES

## Abstract

Adenylyl cyclase (AC) is an important messenger involved in G-protein-coupled-receptor signal transduction pathways, which is a well-known target for drug development. AC is regulated by activated stimulatory (Gα_s_) and inhibitory (Gα_i_) G proteins in the cytosol. Although experimental studies have shown that these Gα subunits can stimulate or inhibit AC’s function in a non-competitive way, it is not well understood what the difference is in their mode of action as both Gα subunits appear structurally very similar in a non-lipidated state. However, a significant difference between Gα_s_ and Gα_i_ is that while Gα_s_ does not require any lipidation in order to stimulate AC, N-terminal myristoylation is crucial for Gα_i_’s inhibitory function as AC is not inhibited by non-myristoylated Gα_i_. At present, only the conformation of the complex including Gα_s_ and AC has been resolved via X-ray crystallography. Therefore, understanding the interaction between Gα_i_ and AC is important as it will provide more insight into the unknown mechanism of AC regulation. This study demonstrates via classical molecular dynamics simulations that the myristoylated Gα_i1_ structure is able to interact with apo adenylyl cyclase type 5 in a way that causes inhibition of the catalytic function of the enzyme, suggesting that Gα lipidation could play a crucial role in AC regulation and in regulating G protein function by affecting Gα_i_’s active conformation.

## Author summary

Communication between cells is essential for the survival of any multicellular organism. When these mechanisms cannot function properly, diseases can occur such as heart failure or Parkinson’s disease. Understanding cell communication is therefore crucial for drug development. Important proteins in cellular signalling are the ones that initiate mechanisms in the cell after the signal of an extracellular trigger is transported from outside to inside the cell. G proteins (GPs) are an example of such proteins. Experimental studies have shown that GPs can perform stimulatory or inhibitory functions, however, it is not well understood what the difference is in their mode of action, especially as they are structurally very similar. Adenylyl cyclase (AC) is an enzyme which can be stimulated or inhibited by GPs, depending on which type of GP is active. Hence, AC is a good candidate for investigating the difference in function between GPs. However, only the structure of the stimulatory GP interacting with AC is known. Here, we investigate for the first time the effect of the interaction of an inhibitory GP with AC via classical molecular dynamics simulations in order to obtain a better understanding of the difference between stimulatory and inhibitory GP association and AC regulation.

## Introduction

Many proteins are involved in cell communication of which one type is the G-protein-coupled receptor (GPCR), embedded in the membrane. GPCRs are part of a major signalling pathway, the GPCR signal transduction pathway, which enables the transfer of a signal from the extracellular region to the intracellular side and is a key target for drug development. A large diversity of GPCRs can be found in nature as about 800 human genes are involved in storing different types of GPCRs that can interact with neurotransmitters, hormones or exogenous ligands, for example [1].

In the cytosol, G proteins, composed of an α, β and γ subunit, are the first interaction partner of activated GPCRs. When a heterotrimeric G protein is activated by a GPCR, the trimer dissociates, resulting in an α subunit and a βγ dimer [2]. Activated Gα subunits transport the signal from the membrane to other regions of the cell by stimulating or inhibiting reactions via protein-protein interactions. Besides direct activation by GPCRs, the function of G proteins can also be influenced by other environmental factors, such as lipidation. Permanent N-myristoylation, for instance, is known to change the structure and function of the inhibitory G-protein subunit Gα_i1_ in its active GTP-bound state [3–6].

**Fig 1:**
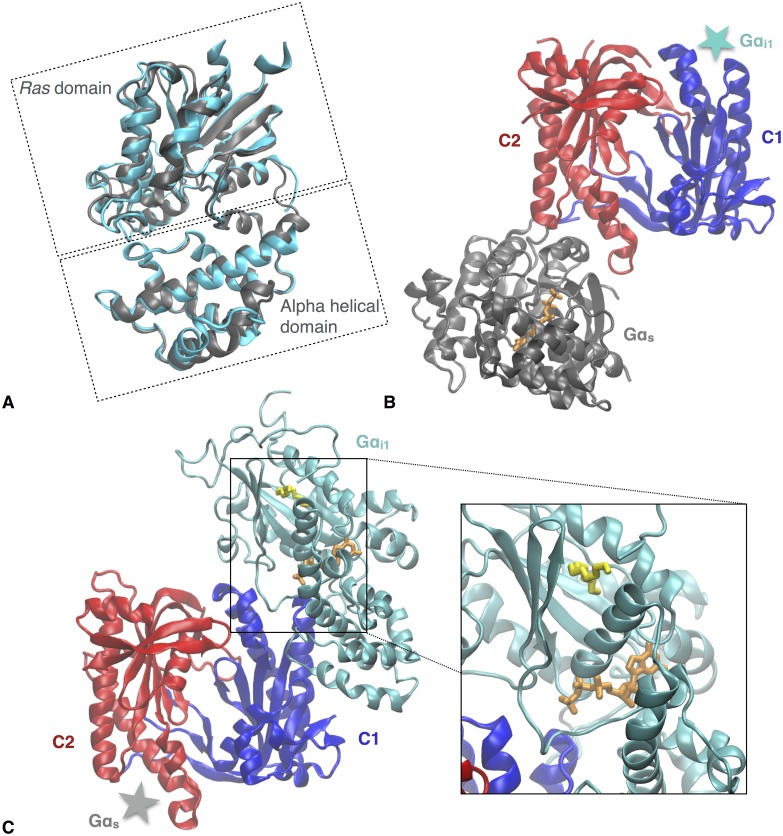
Differences and similarities of Gα:AC complexes and activated Gα subunit structures. (A) Structural alignment of GTP-analog-bound Gα_i1_ (PDB code 1AZT) in cyan and GTP-analog-bound Gα_s_ (PDB code 1AS0) in grey. (B) View of the Gα_s_:AC complex (PDB code 1AZS) from the cytosolic side. The Gα_s_ subunit is depicted in grey, while the C1 domain is represented in blue and the C2 domain is shown in red. The location of the Gα_i1_ structure is described by the cyan star. (C) View of the docked Gα_i1_^myr^:AC5 complex from the cytosolic side. The Gα_i1_^myr^ subunit is depicted in cyan with the myristoyl moiety shown in yellow and the GTP molecule in orange. The C1 domain is represented in blue and the C2 domain is shown in red. The location of the Gα_s_ structure is described by the grey star.

While a wide range of GPCRs exists, a relatively low diversity is present in the G protein family, e.g. in the human body. The human body includes only a relatively small variety of 21 α, 6 β and 12 γ subunits [1]. The Gα subunits are divided into four major subfamilies based on their sequence homology and function [7]: stimulatory Gα_s_, inhibitory Gα_i_, Gα_q_ and Gα_12_ [8, 9]. Overall the structures of the Gα subfamilies are similar (S1 Fig, Fig 1), including a *Ras* domain and an alpha helical (AH) domain. The *Ras* domain is present in all members of the G-protein superfamily and can perform hydrolysis of GTP to GDP during deactivation of the Gα subunit [10]. In addition, the domain includes an interaction site for GPCRs as well as regions that can interact with the βγ dimer. Moreover, the *Ras* domain can also undergo lipidation. Except for Gα_t_, all Gα proteins are able to reversibly bind a palmitate to their N-terminal helix. Besides palmitoylation, Gα_i_ can also permanently bind a myristoyl moiety to the N-terminus that appears to be crucial for the function of the subunit (Fig 1C) [4, 5, 9, 11].

The AH domain is unique to the Gα subfamilies, which is composed of six α helices and interacts with the *Ras* domain when GTP or GDP is present (Fig 1C). However, this interaction between the AH and *Ras* domain is weakened when a nucleotide is absent in Gα’s active site [12–14]. The high structural similarity among members of the Gα subfamilies is illustrated by aligning the X-ray structures of stimulatory Gα_s_ and inhibitory Gα_i1_, resulting in a root mean square deviation (RMSD) of only 1.07 Å between the Cα atoms of the two structures (Fig 1A) [15, 16]. Hence, from a comparison of the structures it is difficult to conclude what the origin is of their inverse action, i.e., how the structure can be related to a stimulatory respectively an inhibitory effect.

An example of a protein in which both Gα_i_ and Gα_s_ are important for regulation is adenylyl cyclase (AC). Ten isoforms of AC are known of which nine are membrane-bound (AC1-9) and one is soluble (sAC) [17]. These different types of AC are found throughout the body in different concentrations. AC5, for instance, is present in high quantities in the brain, the spinal cord and the heart, and is associated to congestive heart failure and pain perception [18, 19]. G proteins have the ability to either stimulate (G_s_) or inhibit (G_i_) adenylyl cyclases’ conversion of adenosine triphosphate (ATP) to cyclic adenosine monophosphate (cAMP) and pyrophosphate [20, 21].

ACs consist of two membrane-bound regions, each built from six trans-membrane domains, and a catalytic region in the cytosol that includes two pseudo-symmetric domains, C1 and C2 (Fig 1) [22]. GTP-bound Gα_s_ is known to bind to the C2 domain for which the interaction site is known from X-ray structures of Gα_s_ interacting with AC (Fig 1B) [23]. Such data is absent for the case of Gα_i_. In the absence of direct experimental information, a putative interaction site of GTP-bound Gα_i_ has been suggested in analogy to the known structure of the complex of Gα_s_ and AC (Gα_s_:AC) as the pseudo-symmetric site on the C1 domain (Fig 1B). However, how the interaction of Gα_i_ on the C1 domain should induce inhibition is not obvious [5]. Furthermore, since with this hypothesis the interaction sites of Gα_i1_ and Gα_s_ are highly similar in addition to their structures, it is unclear how the α subunits can differentiate the two binding sites on AC and what the cause is of the stimulatory versus the inhibitory effect induced by the subunits.

A factor that could play an important role in differentiating the action of Gα_i_ and Gα_s_ is the difference in lipidation of both subunits. Although the X-ray structures in the Protein Data Base (PDB) [24] of the active inhibitory and stimulatory Gα subunits tightly align, the N-terminus, which is not resolved for Gα_i_ or Gα_s_, is not myristoylated during the expression process of Gα_i_ as lipidation can hinder crystallisation [4]. Hence, it is not clear to what extent the missing N-terminal myristoyl moiety affects the Gα_i_ structure of the remaining protein while the bound myristoyl group has been known to be crucial for Gα_i_’s conformation and function as the ability to interact with AC5 is abolished upon removal of the myristate [4–6]. Classical molecular dynamics (MD) simulations of myristoylated GTP-bound Gα_i1_, Gα_i1_^myr^, demonstrate the stability of the myristoyl moiety on the *Ras* domain due to a hydrophobic pocket formed by β2-β3, α1 and the C-terminus α5 (Fig 1C) and show that myristoylation can have a significant effect on the conformation of the subunit [25]. The findings suggest the possibility of an alternative novel interaction mode and open up new possibilities for selective interactions with AC. This is because the found structural changes in the classical MD simulations of Gα_i1_^myr^ [25] suggest that the subunit will not be able to interact with C1 as Gα_s_ interacts with C2.

Here, we investigate the interaction between Gα_i1_^myr^ and AC, using classical MD simulations. To this end, the initial structure of Gα_i1_^myr^ was taken from reference [25] in which a 2 μs classical MD simulation of Gα_i1_^myr^ is described. Gα_i1_^myr^ can inhibit only particular isoforms of AC: AC1, AC5, AC6 [26]. In this study AC5 is used because X-ray structures of AC’s catalytic domains are composed of isoforms AC2 and AC5. Ca. 16 AC structures can be found in the PDB with different resolutions and/or crystallisation conditions. All available structures have been co-crystallised with a Gα_s_ subunit and correspond therefore to stimulated conformations at various levels of activation, depending on the nature of bound cofactors (e.g. cofactor-free complex of AC, substrate-bound AC complex).

When AC5 becomes active, roughly three conformational options are possible: a complex of Gα_s_ and AC5, Gα_s_:AC5, Gα_s_ in complex with ATP-bound AC5, Gα_s_:AC5(ATP), or a complex of Gα_s_ and AC5 bound to the reaction products cAMP and pyrophosphate, Gα_s_:AC5(cAMP). Currently, it is not known which one of these forms is most likely to interact with Gα_i1_^myr^, or if Gα_i1_^myr^ can inhibit all of them. In this study, the structure of the AC5 protein was taken from a crystal structure of the cofactor-free Gα_s_:AC5 complex. This apo AC5 structure was used as it could provide insight into Gα_i1_^myr^’s inhibitory effect on a stimulated conformation of AC5 in the absence of ATP. The selected AC5 structure was employed to build a Gα_i1_^myr^:AC5 complex (Fig 1C) and to explore if the binding of Gα_i1_^myr^ is able to affect the active conformation initially induced by Gα_s_. The absence of ATP in the active site provides the opportunity to investigate Gα_i1_^myr^’s ability to prevent the formation of AC’s fully activated form by altering AC’s conformation unfavourably prior to substrate association. In order to verify which changes are due to the interaction of AC5 with Gα_i1_^myr^ and which alterations are a result of the removal of Gα_s_, a second simulation of AC5, with the Gα_s_ subunit removed, was performed on the same time scale as the Gα_i1_^myr^:AC5 complex.

Hence, in this study the impact of the presence of myristoylated Gα_i_ on the function of AC5 is explored via investigating the conformational features of the Gα_i1_^myr^:AC5 and the free AC5 complex (a system that only includes AC’s catalytic region in solution) in comparison with the Gα_s_:AC X-ray structure. The Gα_i1_^myr^:AC5 complex has been obtained via docking the Gα_i1_^myr^ structure on to the C1 domain of AC5. Already the initial docking results confirm the possible importance of the myristoyl-induced structural changes of Gα_i1_^myr^ as a new interaction mode for Gα_i1_^myr^ could be identified. The comparison of the performed classical MD simulation (2.5 μs) of the Gα_i1_^myr^:AC5 complex and the free AC5 system suggest two possible ways of AC inhibition in its apo form. First, Gα_i1_^myr^ seems to inhibit AC’s conversion of ATP to cAMP by preventing active-site formation as the Gα_i1_^myr^ subunit perturbs the conformation of the active site at the C1/C2 interface. Second, the effect of Gα_i1_^myr^ on the AC structure leads to a closed conformation of the Gα_s_ binding site on C2, decreasing the probability of Gα_s_ association and thus of a counter-balancing re-stimulation of the AC5 activity. Taken together, the observed events lead to a suggestion for a putative Gα_i1_^myr^ inhibition mechanism of apo AC5 in which lipidation is crucial for Gα_i1_^myr^’s function and its protein-protein interactions. Hence, the results of this study provide a possible indication that lipidation could play a significant role in regulating G protein function and therefore could impact signal transduction in G protein mediated pathways [4–6].

## Materials and methods

### Initial structures

The PDB structure 1AZS, including the Gα_s_:AC complex with AC in the apo form, was used as a template, including 1AZS’s C1 and C2 domain, for the initial AC5 structure of Rattus norvegicus (UniprotKB Q04400) [27–29]. The structure of the Rattus norvegicus Gα_i1_^myr^ subunit (UniprotKB P10824) interacting with GTP and Mg^2+^ was taken from reference [25] (S2 and S3 Figs).

### Docking of Gα_i1_^myr^ on AC5

The HADDOCK web server [30] was used for docking ten conformations of Gα_i1_^myr^ on the catalytic domains of AC5 of Rattus norvegicus. The Gα_i1_^myr^ snapshots were extracted at the end of the Gα_i1_^myr^ classical MD trajectory (around 1.9 μs) discussed in reference [25], with a time interval of 0.5 ns. The active region of Gα_i1_ was defined in HADDOCK as a large part of the AH domain (112-167), the switch I region (175-189) and the switch II region (200-220), allowing for a large unbiased area on the Gα_i1_^myr^ protein surface to be taken into account during docking. The active region of AC5’s C1 domain was defined as the α1 helix (479-490) and the C-terminal region of the α3 helix (554-561) because experimentally it has been found that Gα_i1_^myr^ is unable to interact with C2 and its main interactions with AC are with the C1 domain [5]. Passive residues, residues that could take part in protein-protein interaction, were defined as residues around the active residues that are on the protein surface and within a radius of 6.5 Å of any active residue [30].

The initial Gα_i1_^myr^:AC5 complex for the classical MD simulations was selected based on (1) the absence of overlap between the C2 domain and Gα_i1_^myr^, (2) no overlap with Gα_i1_^myr^’s GTP binding region and the interaction site of Gα_i1_^myr^ with C1 and (3) presence of similar complexes in the top-ten docking results of the docking calculations performed for all ten Gα_i1_^myr^ snapshots. The first property of the selection criteria is important since Gα_i1_^myr^ is unable to interact with C2 [5]. The second criterium has been defined since GTP is located in the active site of Gα_i1_^myr^ in the classical MD simulations, but was not incorporated in the docking procedure because this is not possible in HADDOCK. Therefore, no overlap between the GTP binding site and the C1 domain should be present in the docking result as otherwise the GTP molecule will not be able to fit in Gα_i1_^myr^’s active site. The last criterium is the presence of similar Gα_i1_^myr^:AC5 complexes of the selected complex in all top-ten docking results which increases the probability that complexation of the two proteins is not conformation specific, but is robust as similar complexes can be obtained using different conformations of Gα_i1_^myr^.

### Classical molecular dynamics simulations

The Gα_i1_^myr^:AC5 complex was used to simulate the protein complex for 2.5 μs at 310 K and 1 bar using a Nosé-Hoover thermostat and an isotropic Parrinello-Rahman barostat. In the active site of Gα_i1_^myr^ one Mg^2+^ ion and a GTP molecule are present. In order to closer mimic an AC5 system with which ATP or a product such as pyrophosphate would be able to interact, a Mg^2+^ ion was added to the active site of AC5 (see S1 Appendix). Additionally, about 68 000 water molecules and 150 mM KCl are present in the simulated system.

The force fields used for the protein and the water molecules are AMBER99SB [31] and TIP3P [32], which were employed by Gromacs 4.6.6 [33, 34] to perform the runs. For GTP, the force field generated by Meagher *et al.* was used [35]. The adjusted force field parameters for the K^+^ ions and the Cl^-^ ions were taken from Joung *et al.* [36]. The Mg^2+^ ion parameters originated from Allnér *et al.* [37] and the parameter set for the myristoyl group was taken from reference [25]. The charges for the myristoyl group were obtained with Gaussian 09 [38] based on Hartree Fock calculations in combination with a 6-31G* basis set and using the AMBER RESP procedure [39]. Appropriate atom types from the AMBER99SB force field were selected to complete the myristoyl description.

Electrostatic interactions were calculated with the Ewald particle mesh method with a real space cutoff of 12 Å. Bonds involving hydrogen atoms were constrained using the LINCS algorithm [40]. The time integration step was set to 2 fs.

The free AC5 system was simulated with the same setup as the Gα_i1_^myr^:AC5 complex. The system was solvated in 30 000 water molecules and a 150 mM KCl concentration. The initial location of the Mg^2+^ ion in the active site of the enzyme was the same as in the Gα_i1_^myr^:AC5 complex system.

### Structure superpositions and images

Multiprot [41] and VMD [42] were used to align protein structures. Uniprot [43] was used to align protein sequences. Images were prepared with VMD [42].

## Results and discussion

The stability of the docked Gα_i1_^myr^:AC5 complex and the effect of Gα_i1_^myr^ association was verified via investigating the conformational changes in the complex through classical MD simulations on the μs time scale. The X-ray structure used for the initial conformation of AC5 in the simulated complexes, was co-crystallised with Gα_s_ but is not interacting with substrate or products from the ATP conversion reaction (see Introduction). Consequently, this conformation of AC5 could be viewed as a structure that is present before ATP association, but is already in an active form, due to its interactions with Gα_s_. Because of the crystallisation circumstances used to obtain the selected AC5 structure, the found conformational changes in the catalytic region of AC5 in the Gα_i1_^myr^:AC5 complex are compared to the conformation found in the classical MD trajectory of the free AC5 system and the Gα_s_:AC X-ray structure in order to verify which structural alterations are due to the presence of Gα_i1_^myr^ and which changes are the result of the absence of Gα_s_.

### Protein-protein interface of the Gα_i1_^myr^:AC5 complex

**Fig 2:**
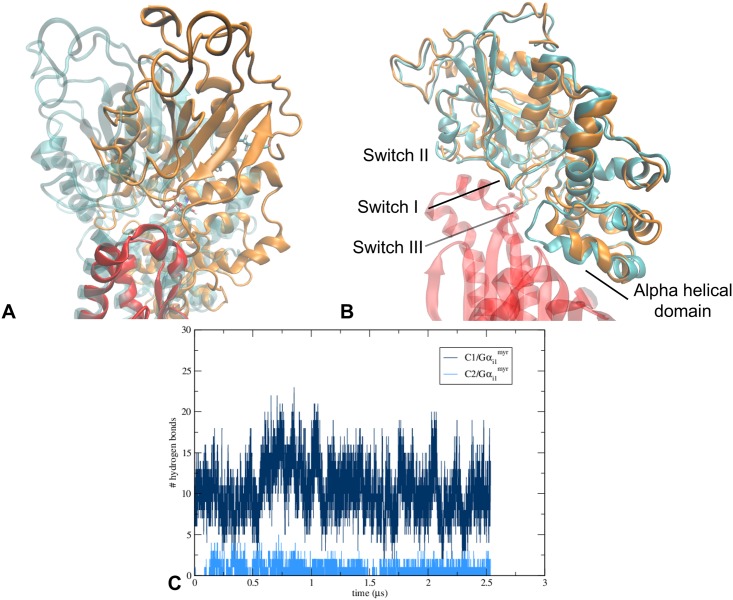
Gα_i1_^myr^’s interactions with AC5 are stable over the course of the classical MD trajectory. (A) Aligned structures of the docked model (cyan) and the Gα_i1_^myr^:AC5 complex after ∼ 1.7 μs (orange and red). The structures were aligned on the C1 domain, residues 456 to 644, as this domain’s RMSD is low over the course of the simulation (Fig 3C). (B) Aligned structures of the docked model (cyan) and the Gα_i1_^myr^:AC5 complex after ∼ 1.7 μs (orange and red). The structures were aligned on the Gα_i1_^myr^ subunit (residues 34 to 334) in order to show the change in the conformation of Gα_i1_^myr^. (C) Number of hydrogen bonds between Gα_i1_^myr^ and C1 and Gα_i1_^myr^ and C2 that are present during the classical MD trajectory.

The initial conformation of the Gα_i1_^myr^:AC5 complex suggests that Gα_i1_^myr^’s proposed interaction site (see S1 Appendix) affects the conformation of C1 in a different way than Gα_s_ stabilises the C2 domain (Figs 1, 2 and S3 Fig). Unlike Gα_s_, Gα_i1_^myr^ is not located between the helices of AC5’s catalytic domain, but appears to clamp the C1 domain into its inactive conformation. Gα_i1_^myr^ is positioned around AC5’s α3, interacting with α1, α2, and α3 via its switch I, II and III region together with the C-terminal domain of αB (Fig 2 and S3 Fig). Since C1’s α1 helix appears to decrease its distance with respect to the C2 domain when an ATP analog, adenosine 5-(α-thio)-triphosphate (ATPαS), is present in the active site (S2 Fig), the interactions between Gα_i1_^myr^ and C1’s α1 in the Gα_i1_^myr^:AC5 complex could suggest that one way by which Gα_i1_^myr^ is able to inhibit ATP’s conversion is by preventing C1’s α1 to rearrange upon ATP binding.

#### The initial Gα_i1_^myr^ conformation is very stable over the entire course of the classical MD trajectory

**Fig 3:**
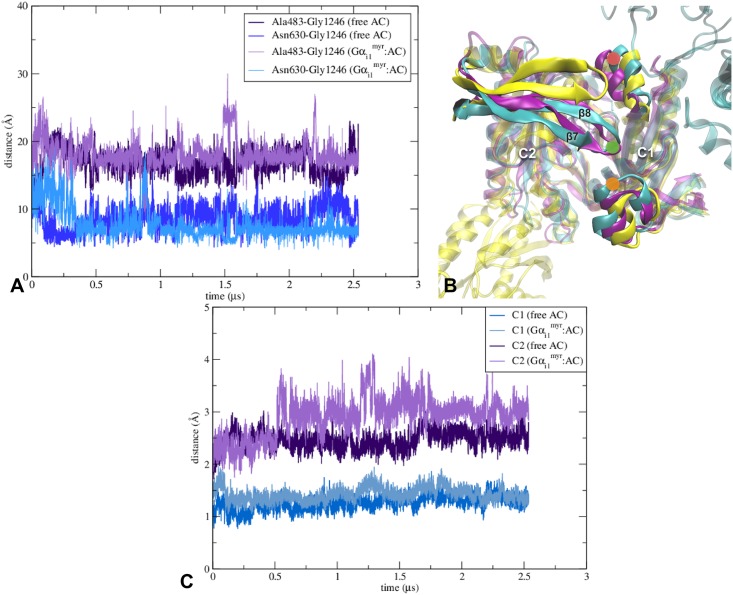
Change of location of C2’s β7-β8 loop occurs in both Gα_i1_^myr^:AC5 and free AC5 systems and a significant difference is observed between RMSD values of free AC5 and Gα_i1_^myr^:AC5 for the C2 domain. (A) Graph of the distances between the Cα atom of Gly1246 (green dot in image *B*) and the Cα atoms of Ala483 (red dot in image *B*) and Asn630 (orange dot in image *B*). (B) β7-β8 loop’s relocation in the free AC5 system (purple), the Gα_i1_^myr^:AC5 complex (cyan) and PDB structure 1AZS (yellow). The location of the residues used in image *A* are assigned according to the Gα_i1_^myr^:AC5 structure. (C) Root-mean-square deviations of the backbone of the C1 and the C2 domain. In the RMSD calculation the residues between 463 to 644 were taken into account for the C1 domain and the residues between 1065 to 1135 and 1145 to 1257 were used for the C2 domain.

Since the starting structure of the Gα_i1_^myr^:AC5 complex is unrelaxed, the conformation of the Gα_i1_^myr^ subunit and the Gα_i1_^myr^/AC5 interface have been investigated during the MD trajectory to study the stability of the Gα_i1_^myr^:AC5 complex and the effect of the AC5 interaction on the Gα_i1_^myr^ structure. In fact, the structure of Gα_i1_^myr^ only changes minimally by a slight adjustment in the orientation of the alpha helical domain (Fig 2B and S6 Fig). A striking feature of the interface between Gα_i1_^myr^ and C1 is the fact that the switch regions, I, II and III, remain involved in AC5 binding, as well as the region on the alpha helical domain that rearranged upon myristoyl binding (Fig 2A and 2B) [25]. Gα_i1_^myr^ is stabilised on AC5 via the C1 domain without major interactions with C2 (Fig 2C). The relative orientation of Gα_i1_^myr^ with respect to the C1 domain stabilises after ∼ 400 ns (S6 Fig). This slight orientational repositioning is probably a consequence of the relocation of C2’s β7-β8 loop, which could be due to the removal of the Gα_s_ subunit from the C2 domain in the initial complex because this β7-β8 loop displacement is present in Gα_i1_^myr^:AC5 as well as in free AC5 (Fig 3).

Besides the orientation of Gα_i1_^myr^, also minor alterations can be observed in Gα_i1_^myr^’s active site. The interactions of the Mg^2+^ ion near the GTP binding site (S7 Fig) are moderately altered compared to the free Gα_i1_^myr^ system [25]. In Gα_i1_^myr^:AC5 both of Asp200’s oxygens, OD1 and OD2, are able to interact with Mg^2+^, while in free Gα_i1_^myr^ only one of Asp200’s oxygens is interacting with the Mg^2+^ ion [25]. The interaction between Mg^2+^ and Asp200’s second oxygen in Gα_i1_^myr^:AC5, OD2, can be temporarily perturbed by a water molecule, which leads to sudden jumps in the Mg^2+^ and Asp200OD2 distance (S7 Fig).

### Structural effect on AC5’s active site

#### Gα_i1_^myr^’s interactions with C1 impact the entrance of the ATP binding site

The active site of AC5 is located at the interface between C1 and C2. Structural changes of both domains upon Gα_i1_^myr^ binding can potentially influence the protein’s activity. When comparing the root-mean-square deviation of the two domains with respect to AC5’s initial conformation, it is clear that in the free AC5 as well as in the Gα_i1_^myr^:AC5 complex the C2 domain experiences more changes than C1 (Fig 3C). In the X-ray structure (PDB code 1AZS) used as a template to construct the initial AC5 structure, the C2 domain is interacting with a Gα_s_ subunit. The removal of Gα_s_ from the C2 domain could affect the RMSD of the domain in the Gα_i1_^myr^:AC5 and free AC5 systems as the initial structure of AC5 is influenced by the presence of Gα_s_.

During the classical MD runs, the first alteration to C2’s initial structure that can be observed in both Gα_i1_^myr^:AC5 and free AC5 is the relocation of the β7-β8 loop, positioned on the cytosolic side (Fig 3). The location of C2’s β7-β8 loop is important for the active conformation of the active site at the C1/C2 interface (Fig 3 and S2 Fig). Loop relocation and the accompanying movement of the two domains appear to have a deactivating effect on the active site (Fig 3 and S2 Fig). For instance, a residue that is known to be part of the active site, Lys1245, located in the β7-β8 loop, is unable to maintain its orientation towards the ATP binding site (S8 Fig). A reason for this conformational change in the Gα_i1_^myr^:AC5 and free AC5 simulations could be the absence of Gα_s_ at the C2 domain that destabilises β7-β8’s location. The presence of Gα_i1_^myr^ appears to increase the stability of the β7-β8 loop relocation compared to the free AC5 system (Fig 3).

**Fig 4:**
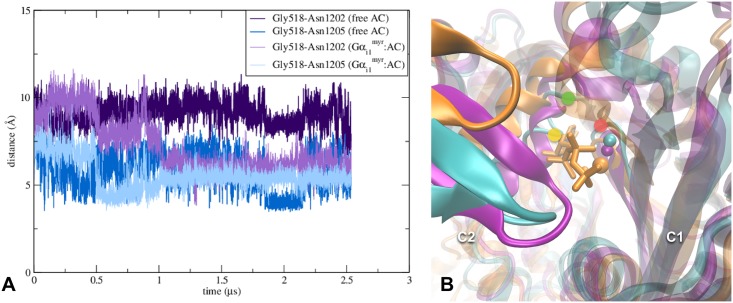
Rearrangements of AC5’s active site differ between the Gα_i1_^myr^:AC5 complex and free AC5 system. (A) Graph of the distances between the Cα atom of Gly518 (red dot in image *B*) and the Cα atoms of Asn1202 (green dot in image *B*) and Asn1205 (yellow dot in image *B*). The respective distances in the Gα_s_:AC(ATPαS) X-ray structure (PDB code 1CJK) of Gly518-Asn1202 (Gly439-Asn1022 in PDB 1CJK) and Gly518-Asn1205 (Gly439-Asn1025 in PDB 1CJK) are 11 Å and 8.5 Å. (B) Detail of AC’s active site of the free AC5 system (purple), the Gα_i1_^myr^:AC5 complex (cyan) and the Gα_s_:AC(ATPαS) X-ray structure (PDB code 1CJK) in orange. The location of the residues used in image *A* are assigned according to the Gα_i1_^myr^:AC5 structure. In the active site the location of the Mg^2+^ ion is shown for all three structures as well as the position of ATPαS from the fully activated AC structure (PDB code 1CJK).

An alteration that also occurs around AC5’s active site in the Gα_i1_^myr^:AC5 system is the decrease in distance between C2’s α4 helix and C1’s β2-β3 loop (Fig 4, S3 Fig). This change in the C1/C2 interface appears to make the positioning of ATP in the active site less favourable because ATP’s adenine moiety, which is positioned between C2’s α4 helix and C1’s β2-β3 loop when ATP interacts with AC5 (S2 Fig), is unlikely to fit between the C1 and the C2 domain due to the diminished distance between C1 and C2 compared to the initial AC5 structure. Although the free AC5 system also undergoes a change in this region, the α4 helix of C2 remains closer to the X-ray location than in the Gα_i1_^myr^:AC5 complex (Fig 4).

In summary, the conformational changes at the C1/C2 interface of Gα_i1_^myr^:AC5 seem to affect AC5’s active site by interfering with ATP binding. In fact, the new position of the β7-β8 loop can even block the active site entrance, which could prevent ATP entry. The relocation of C2’s β7-β8 loop, present in the Gα_i1_^myr^:AC5 and the free AC5 systems, could be due to the removal of Gα_s_, which interacts with the C2 domain in the template X-ray structure. The interaction between Gα_s_ and AC5 seems to stabilise the position of β7-β8 at the C1/C2 interface, close to C1’s α1. As C2’s β7-β8 is part of the active site, the position of β7-β8 could be viewed as a stimulation feature that can be switched on by Gα_s_ or switched off by the absence of Gα_s_ (free AC5) or by the presence of Gα_i1_^myr^, which stabilises the relocation of the β7-β8 loop even more (Fig 3).

#### Gα_i1_^myr^’s interactions with C1 impact AC5’s active site around ATP’s adenine binding site

**Fig 5:**
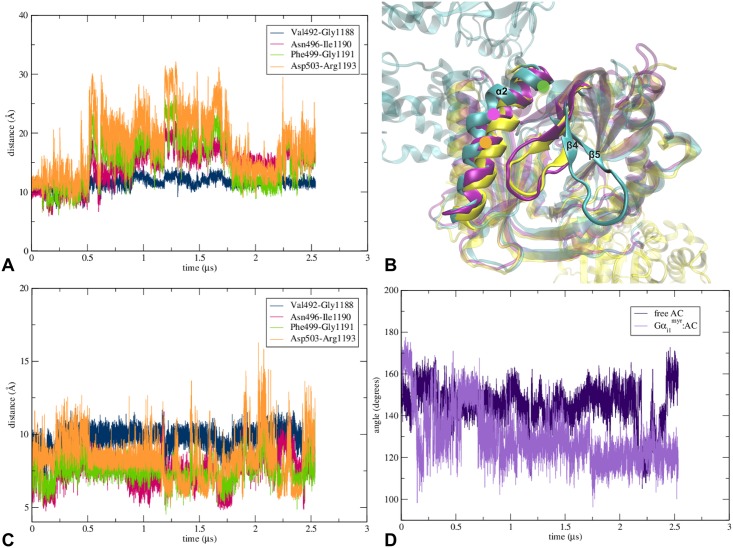
Conformational changes on membrane side of AC5 show C2’s loop dissociation, which only occurs in Gα_i1_^myr^:AC5. (A) Graph of the distances between the α2 helix of C1 and the β4-β5 loop of C2 in the Gα_i1_^myr^:AC5 structure (see image *B*). (B) AC5’s membrane side of the free AC5 system (purple), the Gα_i1_^myr^:AC5 complex (cyan) and PDB structure 1AZS (yellow) in which the α2 helix of C1 and the β4-β5 loop of C2 are highlighted. In the Gα_i1_^myr^:AC5 complex the location of the residues used in the angle calculation of image *D* are represented by a green (Ala488), pink (Leu495) and orange (Phe499) dot. (C) Graph of the distances between the α2 helix of C1 and the β4-β5 loop in the AC5 system (see image *B*). (D) Angle between three helical turns, including Cα atoms of Ala488, Leu495 and Phe499, in which the kinking of the α2 helix of C1 takes place (see image *B*).

While the RMSD of free AC5 and Gα_i1_^myr^:AC5 compared to the initial AC5 structure (obtained via the Gα_s_:AC X-ray structure) appear similar for C1, the C2 domain of Gα_i1_^myr^:AC5 diverges more from the initial structure than free AC5 (Fig 3C). One of the major differences between the C2 domain of the free AC5 system and the Gα_i1_^myr^:AC5 complex lies on the membrane side of the proteins. In the case of free AC5, C2’s β4-β5 loop is interacting with the C1 domain, while in the Gα_i1_^myr^:AC5 complex, the loop does no longer interact with the C1 domain, leading to an unfavourable ATP binding site at the C1/C2 interface (Fig 5A, 5B and 5C). A weakening of the ATP binding site at the C1/C2 interface is also apparent from C1’s interactions with the C2 domain near the active site region (S8 Fig). Asp1198, for example, which is important for stabilising ATP’s adenine moiety in the Gα_s_:AC complex, reorients as the residue is part of C2’s β4-β5 loop. Due to Asp1198’s change in location, Lys1124, which is interacting with Asp1198, alters its orientation as well. Lys1124 also influences the stability of the active site in Gα_s_:AC as the residue stabilises ATP in a similar fashion as Asp1198 (S8 Fig).

Although C1’s RMSD is low (Fig 3C and S6 Fig), a significant change in conformation can be observed close to the Gα_i1_^myr^/C1 interface where a kink in α2 occurs, which is less pronounced in free AC5 (Fig 5D). However, overall, the C2 domain seems to be affected most by the absence of Gα_s_ and the presence of the inhibitory Gα subunit. This observation is in line with the hypothesis that Gα_i1_^myr^ is able to constrain C1’s conformation via its tight interactions with this domain, leading to a perturbation and destabilisation of the active site at the C1/C2 interface (Fig 5 and S7 Fig). This change of the C1/C2 interface prevents the catalytic domains to sample the conformation in which ATP could be positioned in the active site due to a decrease in distance between C2’s α4 helix and C1’s β2-β3 loop and a relocation of C2’s β4-β5 and β7-β8 loops (Figs 3, 4 and 5), which play important roles in the construction of the active site.

#### Presence of Gα_i1_^myr^ induces closure of Gα_s_’s interaction site on AC5’s C2 domain

**Fig 6:**
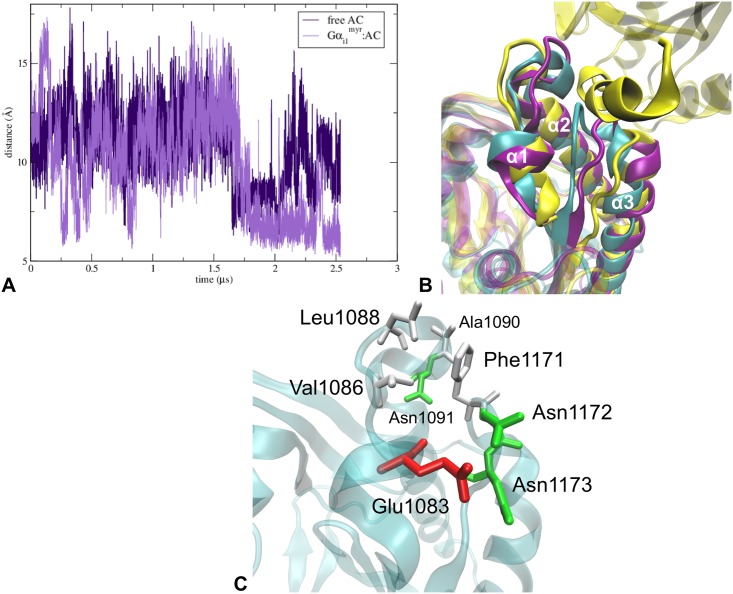
Conformational changes around the Gα_s_ binding site on C2 show distinct events of closure in Gα_i1_^myr^:AC5. (A) Graph of the distance between the α2 and the α3 helix of C2 including Cα atoms of Asn1091 and Phe1171, of free AC5 and Gα_i1_^myr^:AC5. A detailed representation of the Gα_s_ binding site is shown in image *C*. (B) the Gα_s_ binding site of the free AC5 system (purple), the Gα_i1_^myr^:AC5 complex (cyan) and PDB structure 1AZS (yellow) in which the Gα_s_ subunit is also shown. (C) Detail of the Gα_s_ binding site of Gα_i1_^myr^:AC5 in which residues that are involved in the closing of the binding site are shown.

Besides the direct conformational changes in AC5’s active site, another mechanism that could induce inhibition is decreasing the probability of Gα_s_ binding to the C2 domain. In free AC5 as well as in Gα_i1_^myr^:AC5, the Gα_s_ site seems to become less favourable for Gα_s_ binding since the distance between C2’s α2 and α3, α2-α3, is significantly decreased with respect to the X-ray structure of the Gα_s_:AC complex (Fig 6A and 6B). In the free AC5 system α2-α3 changes from around 16 Å (initial distance between the Cα carbons of Asn221 and Phe1171) to an average distance of ∼ 11 Å (Fig 6A and 6B). In the Gα_i1_^myr^:AC5 simulations, the distance between the two residues can decrease even more severely to a distance of ∼ 7 Å (Fig 6A and 6B), leading to a closed Gα_s_ binding site conformation. Important residues that stabilise this closed conformation of the Gα_s_ binding site in Gα_i1_^myr^:AC5 are AC5’s: Glu1083, Leu1088, Ala1090, Phe1171, Asn1172 and Asn1173 (Fig 6D). Hence, due to the interaction with Gα_i1_^myr^, the catalytic domains of AC5 appear to deactivate AC5’s catalytic ability via deformation of the active site as well as by sampling closed Gα_s_ binding site conformations.

### Possible mechanism of Gα_i1_^myr^ inhibition

**Fig 7:**
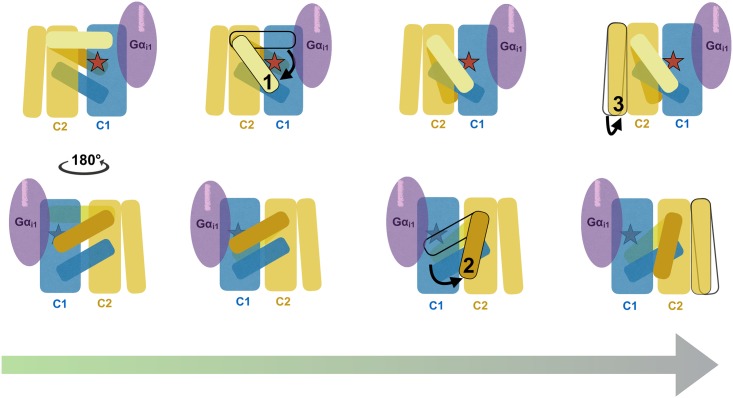
Graphical representation of proposed AC5 inhibition mechanism by Gα_i1_^myr^ with the upper row showing the cytosolic side of AC5 and the bottom row depicting AC5 from the membrane side. The myristoyl moiety bound to Gα_i1_ is shown via a purple line on the subunit. The change that takes place in step (1) compared to the initially stimulated AC5 conformation, is the relocation of the C2 β7-β8 loop away from its active position. This alteration takes place near AC5’s active site (red star), which is also affected by this event. Conformational change (2) involves the loss of interaction between C1’s α2 and the C2 β4-β5 loop, weakening the active site. The final rearrangement (3) includes the closer packing of C2’s Gα_s_ interaction site, which appears to result in a less favourable C2 conformation for the interaction with Gα_s_.

The simulation of Gα_i1_^myr^:AC5 in comparison with the free AC5 trajectory and the Gα_s_:AC X-ray structure demonstrate that the first step in decreasing AC5’s activity in the apo form is the relocation of the β7-β8 loop (Fig 7, step one). In fact, the β7-β8 loop seems to have an important role for the stimulatory response since the presence of Gα_s_ leads to the stabilisation of the loop, forming ATP’s binding site (Fig 7, starting conformation of AC in left panel) [23]. This loop conformation is lost as soon as Gα_s_ is absent, as observed for both free AC5 and Gα_i1_^myr^:AC5. In step two of Fig 7 the Gα_i1_^myr^:AC5 complex undergoes a rearrangement in the C2 domain (absent in free AC5), which leads to a further perturbation of AC5’s active site. The classical molecular dynamics simulations also show that in the presence of Gα_i1_^myr^, there appears to be a decrease in probability for Gα_s_ association (Fig 7, step 3 and Fig 6). Hence, through these rearrangements Gα_i1_^myr^ could deactivate apo AC5 as well as decrease the probability of reactivation via Gα_s_.

### Conclusion

The results of this study suggest that Gα_i1_^myr^ deactivates the apo form of adenylyl cyclase type 5 via constraining C1’s active site region. Inhibition and stimulation of AC5 appear to follow different pathways. While Gα_s_ binds between the helices of C2, increasing the stability of the C1:C2 dimer, Gα_i1_^myr^ is able to clamp the helices of the C1 domain, promoting an inactive conformation of AC5’s catalytic domains and a possible decrease in affinity for Gα_s_ on the C2 domain. Structurally, Gα_s_ and non-myristoylated Gα_i1_ are very similar, however, when myristoylation has taken place on the N-terminus of Gα_i1_, the conformation of the subunit changes drastically, leading to a structure that differentiates itself from the active Gα_s_ subunit and enables the protein to function in an inhibitory fashion as is shown via the presented classical MD simulations. Hence, in line with experimental studies, myristoylation appears to be crucial for G_i_’s function and demonstrates how important even relatively small changes to a protein structure can be for its function.

## Supporting information

S1 Fig Sequence alignment of human Gα_i1_, Gα_i2_, Gα_i3_, which shows a 84% sequence identity between the subunits. (TIF)

S2 Fig Gα_i1_^myr^ interacting with AC type 5 shows a different type of interaction mode with AC5 than Gα_s_. (A) Representation of the docked Gα_i1_^myr^:AC5 complex of Rattus norvegicus with the location of Gα_s_ depicted as well to show the difference in association between the two Gα subunits. Gα_s_ is depicted in gray, Gα_i1_^myr^ in cyan, the C1 domain in blue and the C2 domain in red. The location of the GTP molecules in both Gα subunits is represented by the red pentagons. (B) View from the cytosolic side on the docked Gα_i1_^myr^:AC5 complex, showing the position of the ATP molecule in the catalytic C1 domain in green. The colour scheme is the same as in image A. (C, D) The difference in active AC conformation is shown, depending on AC’s interaction partners (e.g. analog of the substrate ATP, the inhibitor Ca^2+^, substrate free). The alignment of the initial Gα_i1_^myr^:AC5 complex with three AC:Gα_s_ complexes demonstrates that the initial apo AC5 structure used in this study is different from the fully active AC conformation (yellow) to which ATPαS is bound. The yellow structure is a complex of Gα_s_:AC with ATPαS and forskolin (PDB code 1CJK). ATPαS is depicted in transparent yellow in which oxygen atoms are red, phosphor is tan and carbon is cyan. This structure is an active conformation of the AC catalytic domain. The blue (PDB code 3MAA) [44] is suggested to be an inactive Gα_s_:AC complex and interacts with methylpiperazinoforskolin (FKP) together with ATPαS and a Ca^2+^ ion. The red structure (PDB code 1AZS), used as main template for the AC5 conformation in this study, is an Gα_s_:AC complex that only interacts with FKP in the catalytic domain and is more similar to the 3MAA structure than the fully active 1CJK structure around AC’s active site. (E) Alignment of the Gα_i1_^non^:RGS4 complex (PDB code 1AGR) and Gα_i1_^myr^. In case of the non-myristoylated Gα_i1_:RGS4 structure (Gα_i1_^non^:RGS4), Gα_i1_^non^ is shown in red and RGS4 is shown in orange. The myristoylated Gα_i1_ is depicted in cyan. The location of Thr182, Glu207 and Lys210 are shown for both complexes as these residues are important for the interaction between RGS4 and Gα_i1_ [45]. The Gα_i1_^non^:RGS4 residues are labeled in red and the Gα_i1_^myr^ residues are labeled in blue.
(TIF)

S3 Fig View of the docked Gα_i1_^myr^:AC5 complex from the cytosolic side. The Gα_i1_^myr^ subunit is depicted in cyan, while the C1 domain is represented in blue and the C2 domain is shown in red. The location of the Gα_s_ structure is described by the grey star and the GTP molecule is represented by the red pentagon.
(TIF)

S4 Fig Distances between the Mg^2+^ ion and residues in the active site of AC. (A) Distances between the Mg^2+^ ion and residues in the active site of the Gα_i1_^myr^:AC5 system. (B) Distances between the Mg^2+^ ion and residues in the active site of the free AC5 system.
(TIF)

S5 Fig Mg^2+^ ion in the active site of AC of the Gα_i1_^myr^:AC5 complex is located in the same interaction position as in ATPαS or pyrophosphate bound X-ray structures of Gα_s_:AC complexes. (A) Alignment of the Gα_i1_^myr^:AC5 complex (cyan), the free AC5 system (purple) and catalytically active AC (PDB code 1CJK) in orange, which is interacting with ATPαS, showing the active site of AC at the C1/C2 interface. The residue names are following the Rattus norvegicus numbering for AC5. (B) Alignment of the Gα_i1_^myr^:AC5 complex (cyan), the AC system (purple) and AC associated to pyrophosphate, PPi, (PDB code 3C15) in blue showing the active site of AC at the C1/C2 interface. The residue names are following the Rattus norvegicus numbering for AC5.
(TIF)

S6 Fig Root-mean-square deviations of the backbone of the C1:C2 dimer. Additionally also the RMSD of the Gα_i1_^myr^ subunit in the Gα_i1_^myr^:AC5 is shown, together with the RMSD of the combination of Gα_i1_^myr^ and the C1 domain. In the RMSD calculation the residues between 463 to 644 were taken into account for the C1 domain, residues between 1065 to 1135 and 1145 to 1257 were used for the C2 domain and residues 34 to 334 were included for the Gα_i1_^myr^ subunit.
(TIF)

S7 Fig Graph of the distances between the Mg^2+^ ion in the active site of Gα_i1_^myr^. Distances are shown between the Mg^2+^ ion and its environment, including GTP and the Gα_i1_^myr^ residues that are coordinating to the Mg^2+^ ion: Ser47, Asp200.
(TIF)

S8 Fig Changes in the location of important residues in the active site of AC. (A) Detail of the active site of AC in the Gα_i1_^myr^:AC5 complex, showing the residues that are used in the distance calculations for image b and d. Additionally, the position of the Mg^2+^ ion is shown in pink. (B) Graph of the distances in the Gα_i1_^myr^:AC5 system between the Cα carbon of Asp475, which is positioned close to the ATP binding site, and other important residues for ATP conversion: Lys1124, Asp1198, Arg1209 and Lys1245. (C) Detail of the active site of AC in the Gα_s_:AC complex to which ATPαS is bound (PDB code 1CJK), showing the equivalent residues of the residues in AC5 that are used in the distance calculations for image B and D. (D) Graph of the distances in the AC system between the Cα carbon of Asp475, which is positioned close to the ATP binding site, and other important residues for ATP conversion: Lys1124, Asp1198, Arg1209 and Lys1245.
(TIF)

S1 Appendix Supporting results and discussion. (PDF)
