# Supplementary figures and images for: Exploring the inhibition mechanism of adenylyl cyclase type 5 by n-terminal myristoylated Gαi1
Source: PLoS Comput Biol. 2017 Sep 11;13(9):e1005673. doi: 10.1371/journal.pcbi.1005673 (PMC5608429; doi:10.1371/journal.pcbi.1005673)

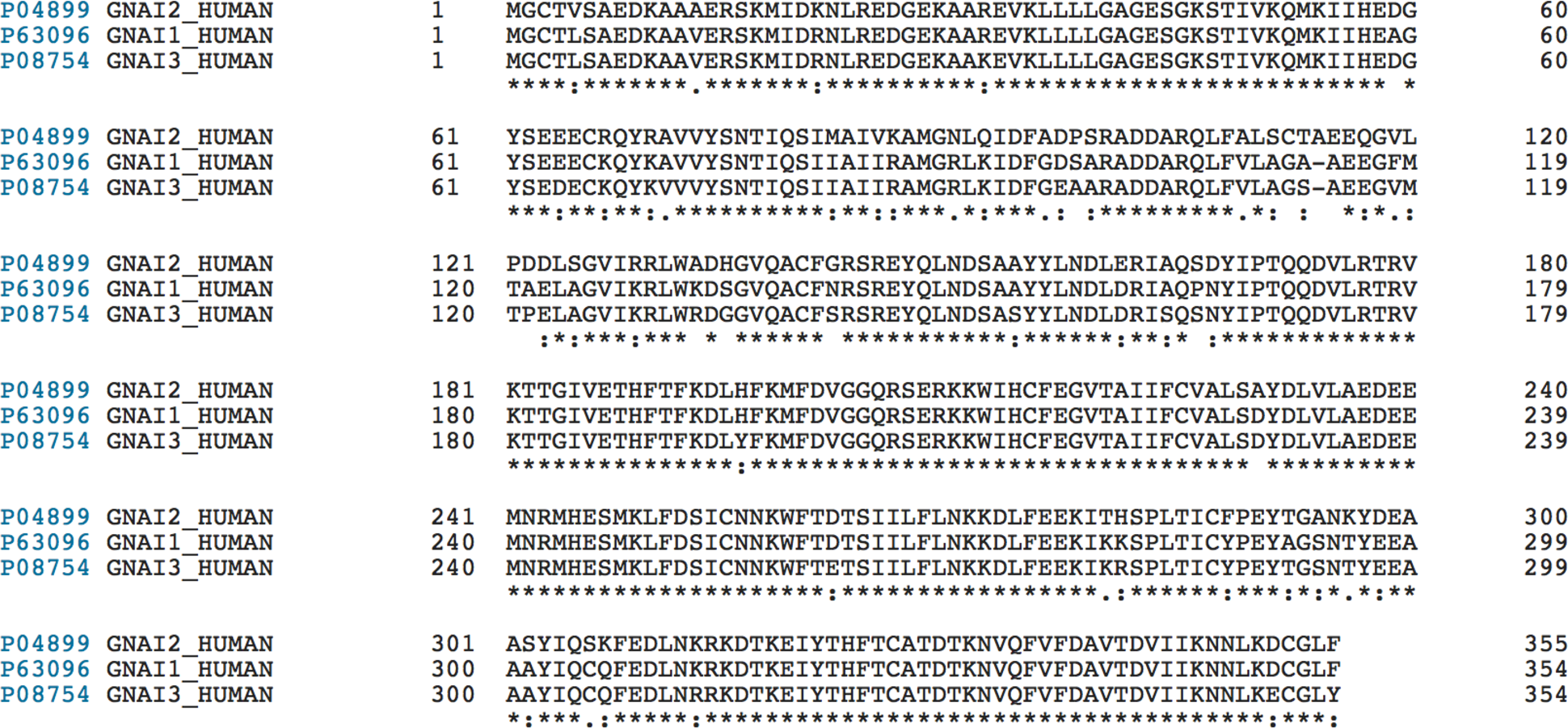

Supplement: S1 Fig — (TIF) [file pcbi.1005673.s001.tif]

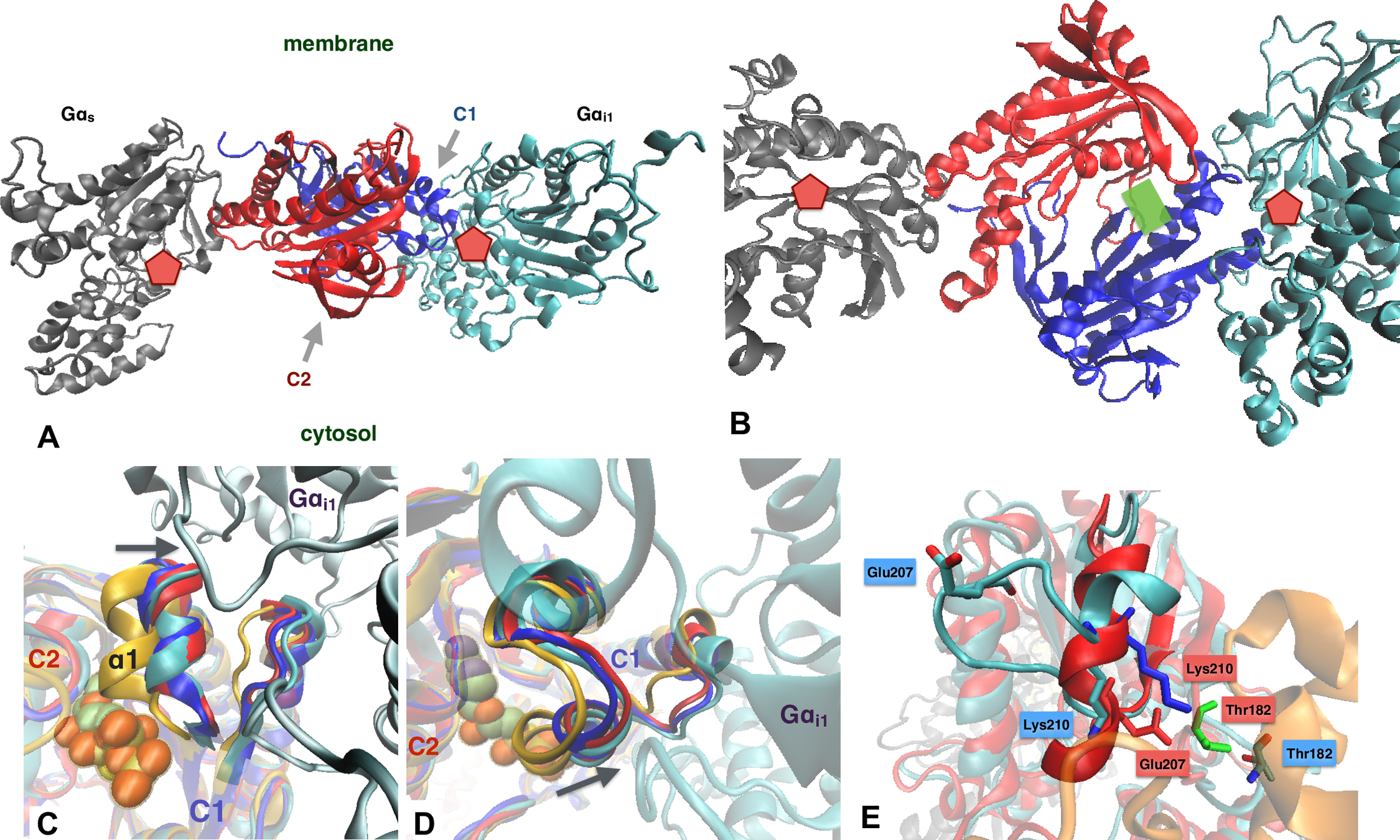

Supplement: S2 Fig — (A) Representation of the docked Gαi1myr:AC5 complex of Rattus norvegicus with the location of Gαs depicted as well to show the difference in association between the two Gα subunits. Gαs is depicted in gray, Gαi1myr in cyan, the C1 domain in blue and the C2 domain in red. The location of the GTP molecules in both Gα subunits is represented by the red pentagons. (B) View from the cytosolic side on the docked Gαi1myr:AC5 complex, showing the position of the ATP molecule in the catalytic C1 domain in green. The colour scheme is the same as in image A. (C, D) The difference in active AC conformation is shown, depending on AC’s interaction partners (e.g. analog of the substrate ATP, the inhibitor Ca2+, substrate free). The alignment of the initial Gαi1myr:AC5 complex with three AC:Gαs complexes demonstrates that the initial apo AC5 structure used in this study is different from the fully active AC conformation (yellow) to which ATPαS is bound. The yellow structure is a complex of Gαs:AC with ATPαS and forskolin (PDB code 1CJK). ATPαS is depicted in transparent yellow in which oxygen atoms are red, phosphor is tan and carbon is cyan. This structure is an active conformation of the AC catalytic domain. The blue (PDB code 3MAA) [44] is suggested to be an inactive Gαs:AC complex and interacts with methylpiperazinoforskolin (FKP) together with ATPαS and a Ca2+ ion. The red structure (PDB code 1AZS), used as main template for the AC5 conformation in this study, is an Gαs:AC complex that only interacts with FKP in the catalytic domain and is more similar to the 3MAA structure than the fully active 1CJK structure around AC’s active site. (E) Alignment of the Gαi1non:RGS4 complex (PDB code 1AGR) and Gαi1myr. In case of the non-myristoylated Gαi1:RGS4 structure (Gαi1non:RGS4), Gαi1non is shown in red and RGS4 is shown in orange. The myristoylated Gαi1 is depicted in cyan. The location of Thr182, Glu207 and Lys210 are shown for both complexes as these residues are important for the [file pcbi.1005673.s002.tif]

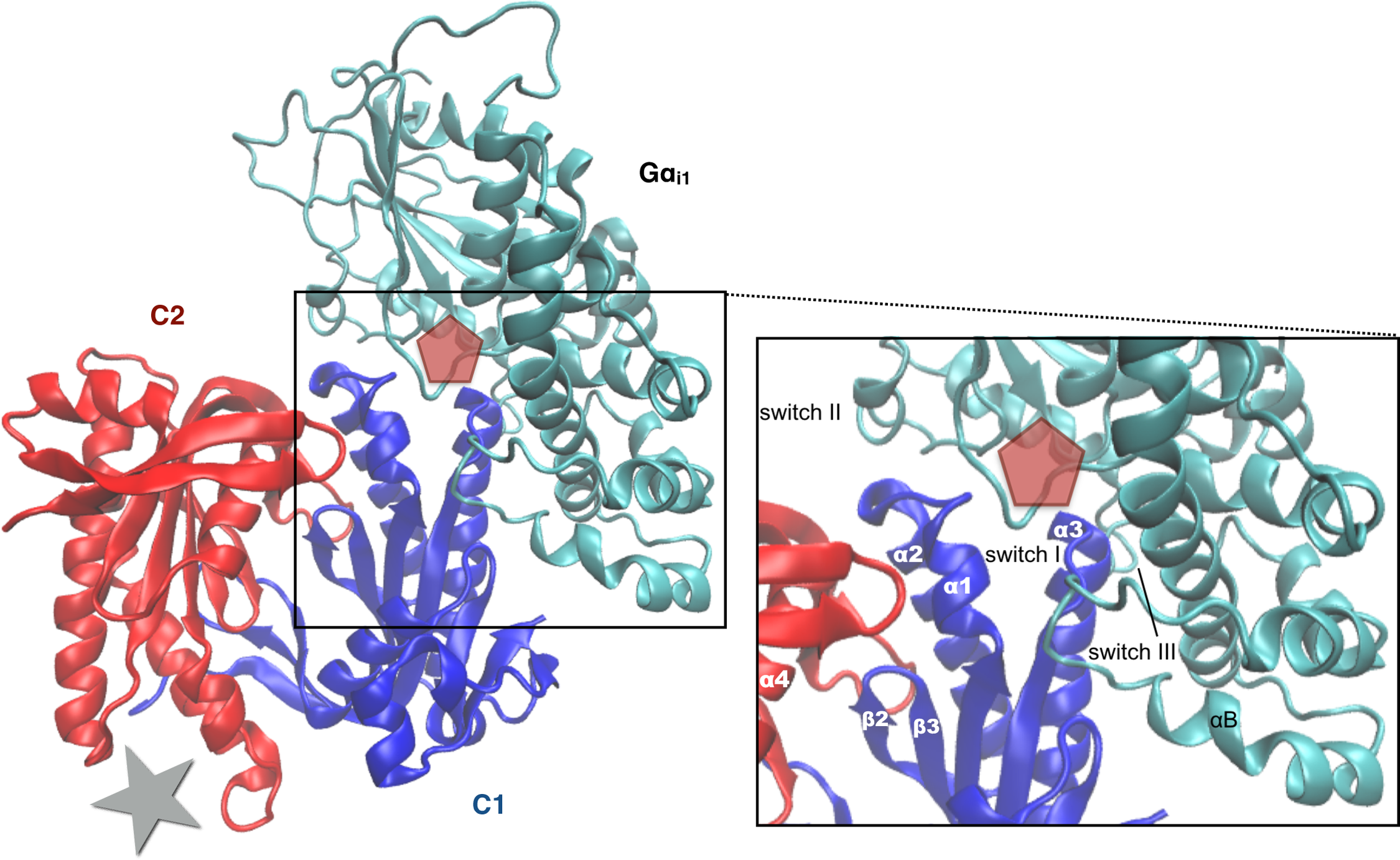

Supplement: S3 Fig — The Gαi1myr subunit is depicted in cyan, while the C1 domain is represented in blue and the C2 domain is shown in red. The location of the Gαs structure is described by the grey star and the GTP molecule is represented by the red pentagon. (TIF) [file pcbi.1005673.s003.tif]

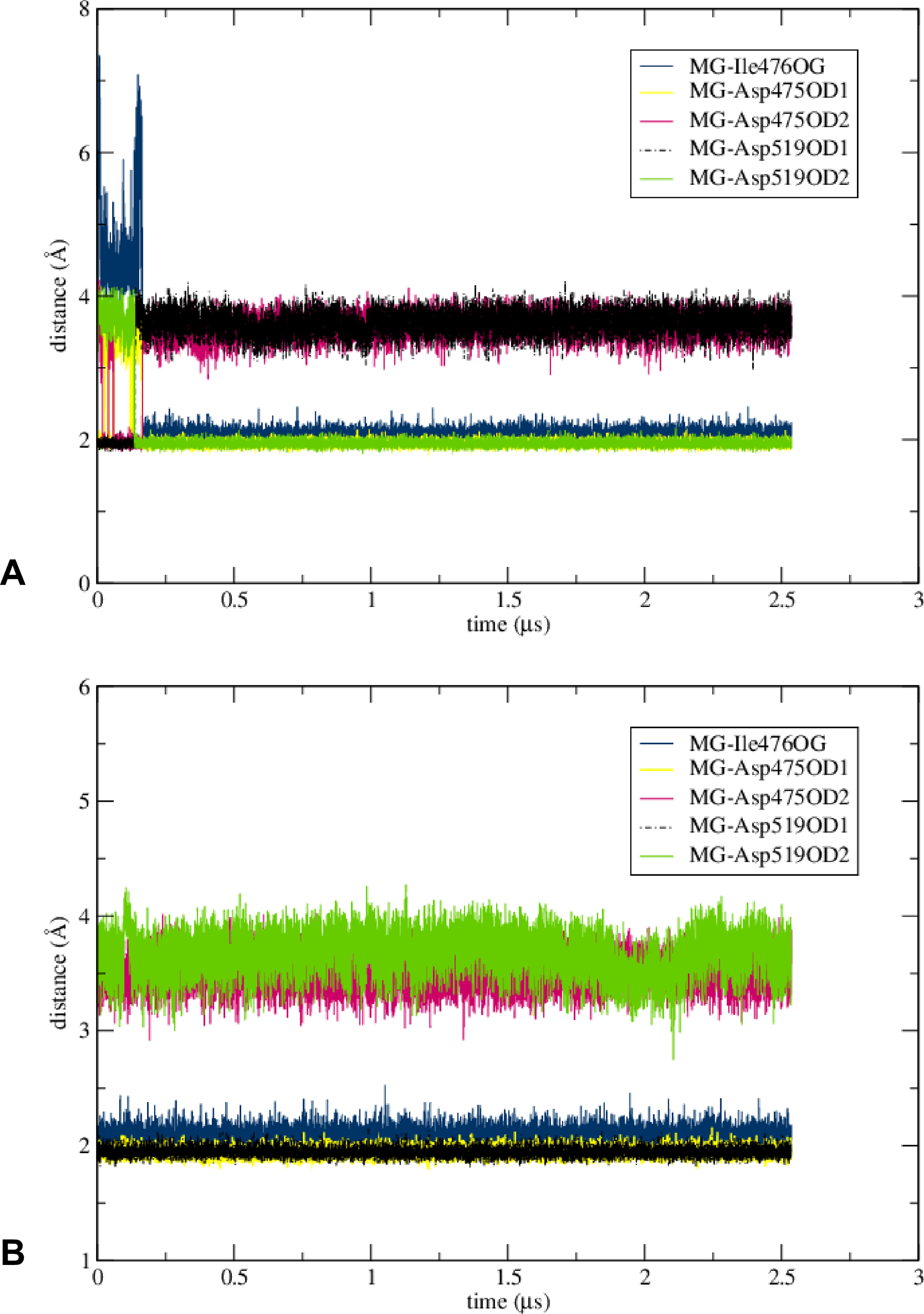

Supplement: S4 Fig — (A) Distances between the Mg2+ ion and residues in the active site of the Gαi1myr:AC5 system. (B) Distances between the Mg2+ ion and residues in the active site of the free AC5 system. (TIF) [file pcbi.1005673.s004.tif]

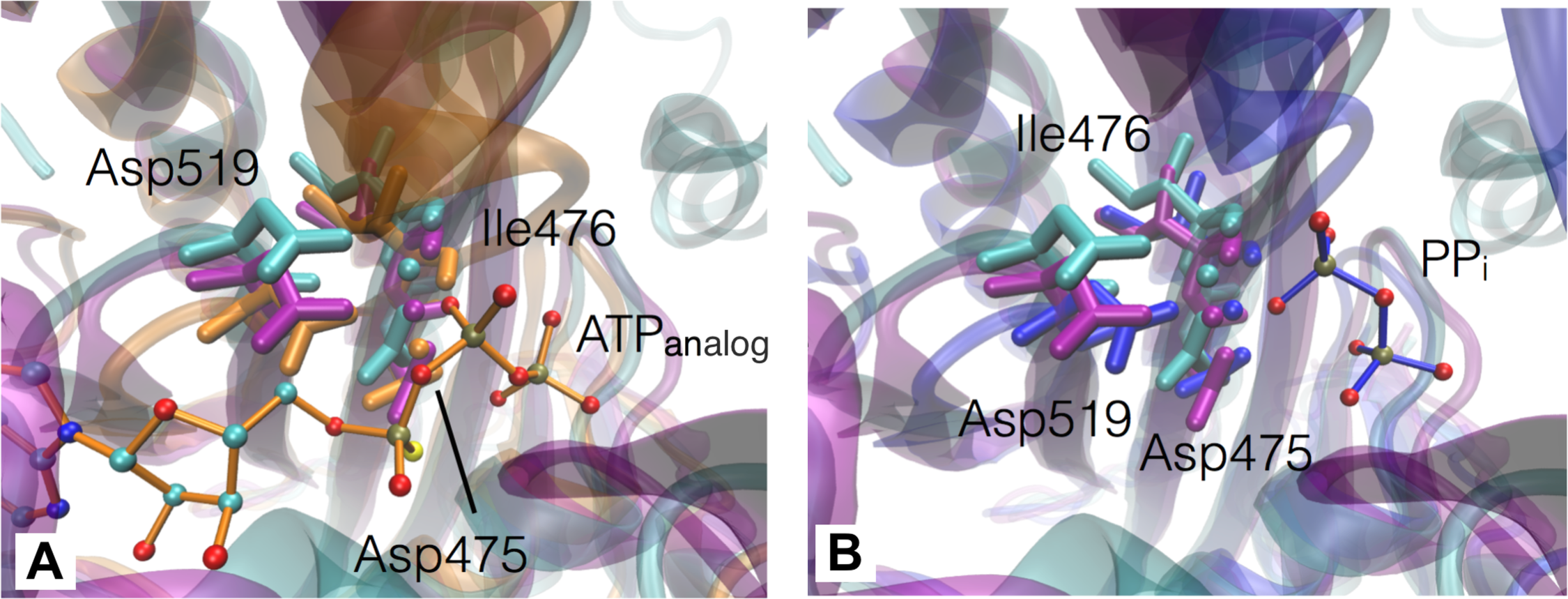

Supplement: S5 Fig — (A) Alignment of the Gαi1myr:AC5 complex (cyan), the free AC5 system (purple) and catalytically active AC (PDB code 1CJK) in orange, which is interacting with ATPαS, showing the active site of AC at the C1/C2 interface. The residue names are following the Rattus norvegicus numbering for AC5. (B) Alignment of the Gαi1myr:AC5 complex (cyan), the AC system (purple) and AC associated to pyrophosphate, PPi, (PDB code 3C15) in blue showing the active site of AC at the C1/C2 interface. The residue names are following the Rattus norvegicus numbering for AC5. (TIF) [file pcbi.1005673.s005.tif]

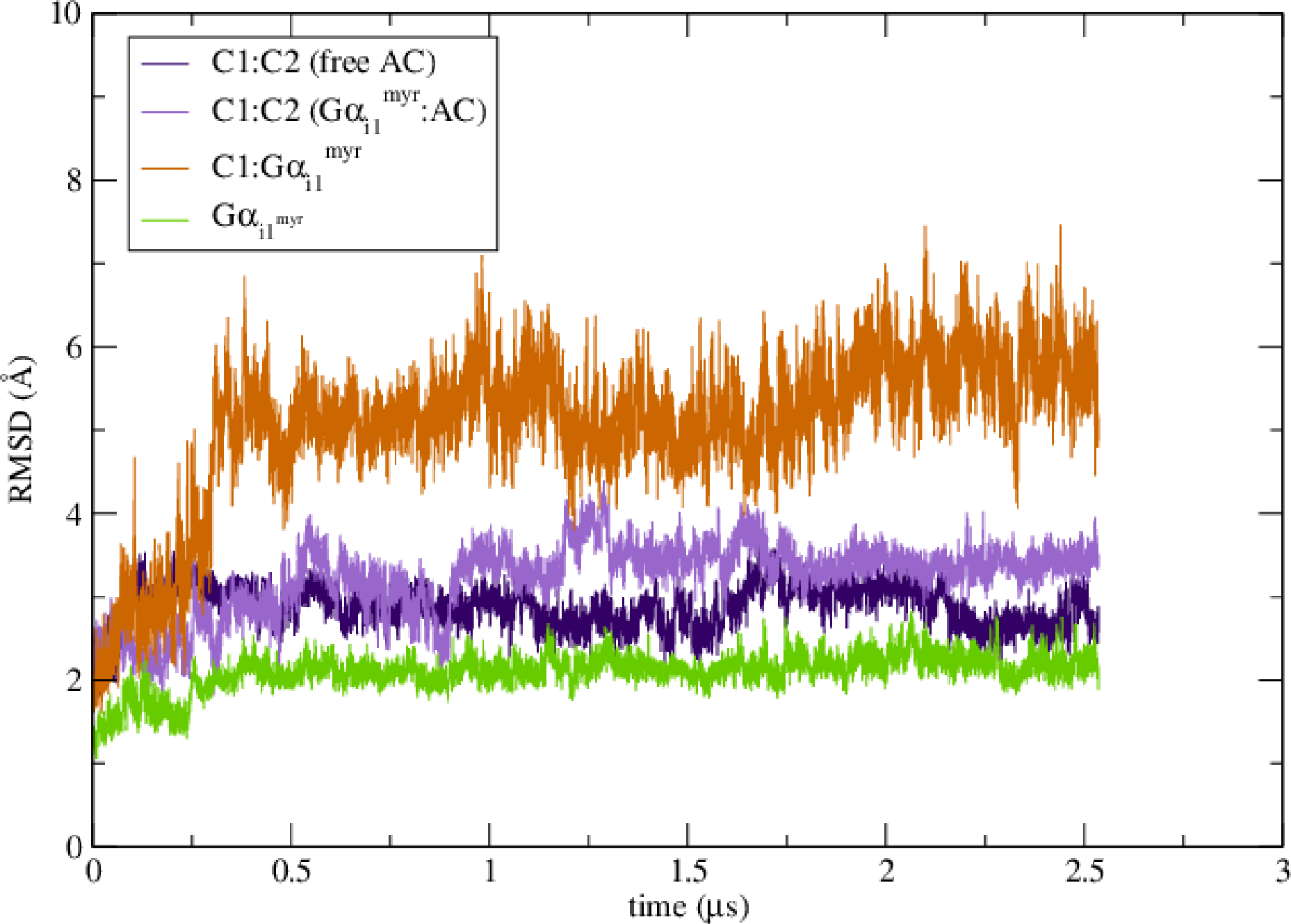

Supplement: S6 Fig — Additionally also the RMSD of the Gαi1myr subunit in the Gαi1myr:AC5 is shown, together with the RMSD of the combination of Gαi1myr and the C1 domain. In the RMSD calculation the residues between 463 to 644 were taken into account for the C1 domain, residues between 1065 to 1135 and 1145 to 1257 were used for the C2 domain and residues 34 to 334 were included for the Gαi1myr subunit. (TIF) [file pcbi.1005673.s006.tif]

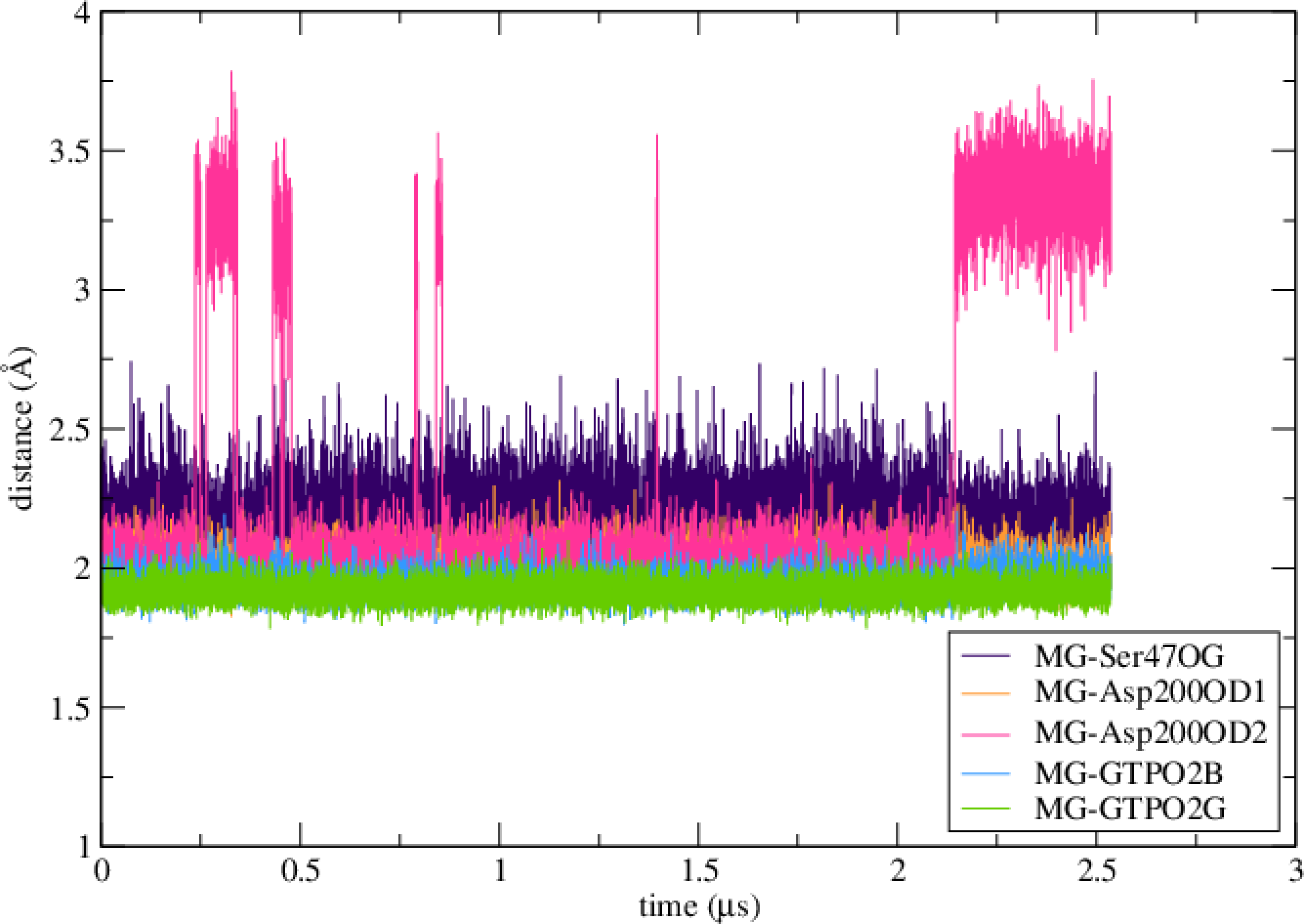

Supplement: S7 Fig — Distances are shown between the Mg2+ ion and its environment, including GTP and the Gαi1myr residues that are coordinating to the Mg2+ ion: Ser47, Asp200. (TIF) [file pcbi.1005673.s007.tif]

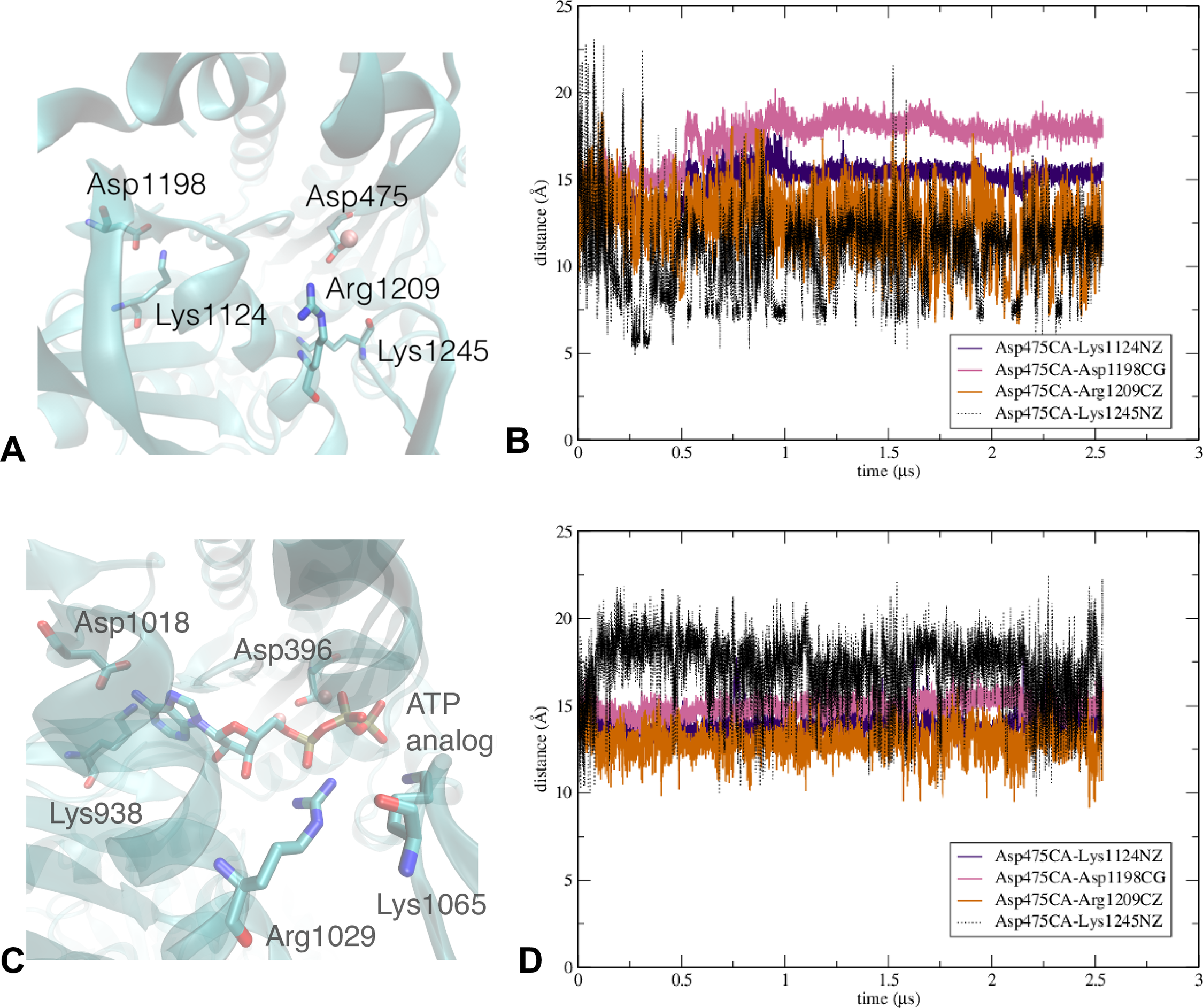

Supplement: S8 Fig — (A) Detail of the active site of AC in the Gαi1myr:AC5 complex, showing the residues that are used in the distance calculations for image b and d. Additionally, the position of the Mg2+ ion is shown in pink. (B) Graph of the distances in the Gαi1myr:AC5 system between the Cα carbon of Asp475, which is positioned close to the ATP binding site, and other important residues for ATP conversion: Lys1124, Asp1198, Arg1209 and Lys1245. (C) Detail of the active site of AC in the Gαs:AC complex to which ATPαS is bound (PDB code 1CJK), showing the equivalent residues of the residues in AC5 that are used in the distance calculations for image B and D. (D) Graph of the distances in the AC system between the Cα carbon of Asp475, which is positioned close to the ATP binding site, and other important residues for ATP conversion: Lys1124, Asp1198, Arg1209 and Lys1245. (TIF) [file pcbi.1005673.s008.tif]
