# Supplementary material for: Exploring the inhibition mechanism of adenylyl cyclase type 5 by n-terminal myristoylated Gαi1
Source: PLoS Comput Biol. 2017 Sep 11;13(9):e1005673. doi: 10.1371/journal.pcbi.1005673 (PMC5608429; doi:10.1371/journal.pcbi.1005673)
Supplement: S1 Appendix — (PDF) [file pcbi.1005673.s009.pdf]

Supporting Results and Discussion

Docking of $G\alpha_{i1}^{myr}$ to AC5

$G\alpha_{i1}^{myr}$:AC5 Complex Suggests a Tighter Binding Mode of $G\alpha_{i1}^{myr}$ on AC Compared to $G\alpha_s$. Image (a) and (b) of S3 Fig. depict the conformation of the initial $G\alpha_{i1}^{myr}$:AC5 structure, used in the classical MD simulation. In order to show the difference in orientation between $G\alpha_{i1}^{myr}$ on C1 and $G\alpha_s$ on C2, $G\alpha_s$'s location (as observed in the crystal structure) is also depicted in the figure. The selected complex from the docking results is characterised by high surface overlap with the C1 domain and a non-existing overlap with the C2 domain (S4 Fig.), which is consistent with the experimental data of Dessauer *et al.* [5]. $G\alpha_{i1}^{myr}$ also covers the experimentally proposed pseudo-symmetric $G\alpha_{i1}$:AC binding site on the C1 domain of AC5, the cleft formed by helices α2 and α3 [5] (S4 Fig.). Yet, unlike the suggested experimental mode of interaction, $G\alpha_{i1}^{myr}$ does not bind within the α2-α3 cleft, but clamps around the helices, forming a tight mode of interaction with AC5 (S2 Fig. and S4 Fig.).

An additional validation of the proposed model of AC5 and $G\alpha_{i1}^{myr}$ is the comparison of active $G\alpha_{i1}^{myr}$ and the complex of non-myristoylated $G\alpha_{i1}$ ($G\alpha_{i1}^{non}$) and RGS4 as it has been suggested that the $G\alpha_{i1}^{myr}$:AC complex is insensitive to RGS4, a regulator of G-protein signalling (S3 Fig.) [21]. When comparing the $G\alpha_{i1}^{myr}$/AC and the $G\alpha_{i1}^{non}$/RGS4 interface (PDB code 1AGR) [45], it is clear that residues in the switch II and switch I region of $G\alpha_{i1}^{myr}$ that are important for RGS4 binding, such as Thr182 and Glu210, are shifted compared to $G\alpha_{i1}^{myr}$ in the RGS4 complex (S3 Fig.). Since AC5's C1 domain is interacting with and therefore restraining the conformation of $G\alpha_{i1}^{myr}$, the solvated $G\alpha_{i1}^{myr}$ appears to be incompatible with RGS4 binding due to the shift in the switch I and switch II region. This shift could be a reason for $G\alpha_{i1}^{myr}$'s insensitivity to RGS4 when complexed to AC.

Mg^{2+} in AC's Active Site Migrates to a Position also Found in Substrate and Product Bound States

The initial location of the Mg^{2+} ion in the active site was chosen close to the known Mg^{2+} sites to study whether the ion would migrate to one of the locations known via X-ray crystallography. Indeed a relocation of the Mg^{2+} ion occurs in both systems (free AC and the $G\alpha_{i1}^{myr}$:AC complex) to a common Mg^{2+} binding site, which is occupied in ATPαS and pyrophosphate bound forms of AC (S5 Fig. and S6 Fig.). Throughout the MD simulations of free AC5 and $G\alpha_{i1}^{myr}$:AC5, the Mg^{2+} ion remains stably bound to this region of AC5's active site.
